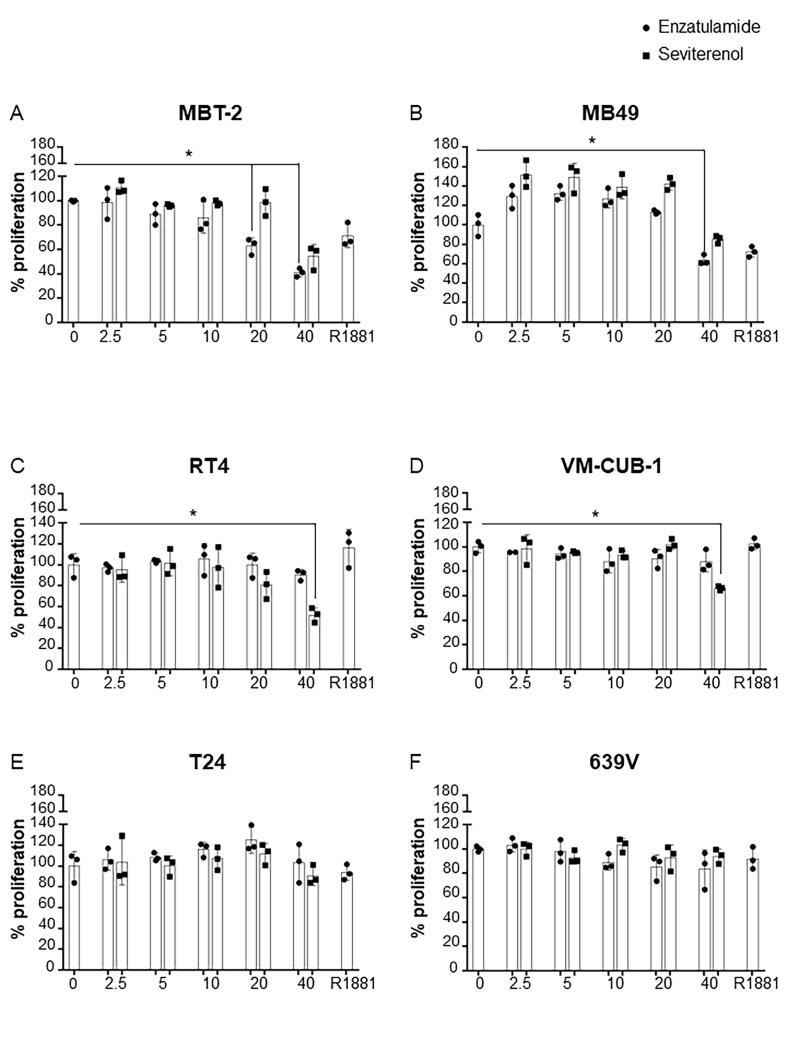# Combining Antiandrogens with Immunotherapy for Bladder Cancer Treatment

**DOI:** 10.1016/j.euros.2022.06.007

**Published:** 2022-07-26

**Authors:** Marjorie Besançon, Typhaine Gris, France-Hélène Joncas, Valérie Picard, Alain Bergeron, Yves Fradet, Paul Toren

**Affiliations:** a Laboratoire d’Uro-Oncologie Expérimentale, Centre de recherche du CHU de Québec-Université Laval, Axe Oncologie, Québec, QC, Canada; b Centre de recherche sur le cancer de l’Université Laval, Québec, QC, Canada; c Département de chirurgie, Université Laval, Québec, QC, Canada

**Keywords:** Bladder cancer, Antiandrogens, Immunotherapy, MBT-2 model

## Abstract

**Background:**

Men are three to four times more likely to be diagnosed with bladder cancer (BCa) than women, who often have more aggressive tumors. Intravesical bacillus Calmette-Guerin (BCG) for non–muscle-invasive bladder cancer (NMIBC) is one of the first immunotherapies, with use of immune checkpoint inhibitors for BCa immunotherapy expanding. Sex hormones, and notably androgens, might impact the outcome of these therapies.

**Objective:**

To understand immunological sex differences in BCa and investigate androgen receptor (AR) inhibition as a novel strategy to improve the response to BCa immunotherapy.

**Design, setting, and participants:**

Human NMIBC tumors were freshly collected following transurethral resection. *In vivo* studies used the subcutaneous MBT-2 BCa model in male and female C3H mice. The AR antagonist enzalutamide was given alone or in combination with anti–programmed cell death protein-1 (anti–PD-1) or intratumoral BCG + poly(I:C) treatments.

**Outcome measurements and statistical analysis:**

Tumor growth and survival were evaluated *in vivo*. Flow cytometry and RNA sequencing characterized the immune cells present in murine and human tumors. Descriptive comparisons were performed for MBT-2 tumors between sexes and with human NMIBC tumors.

**Results and limitations:**

The MBT-2 model shows multiple similarities to the immune composition of human NMIBC tumors and recapitulates previously observed human tumor immune cell sex differences. Enzalutamide in combination with either anti–PD-1 or BCG + poly(I:C) treatment in male mice synergized to improve response rates. Notably, the proportion of complete responses in male mice treated with the combination treatment resembles that observed in female mice with either immunotherapy alone. Limitations include the sample size for murine experiments.

**Conclusions:**

Our results suggest that combining AR antagonism with immunotherapy in male BCa patients may potentiate the antitumor immune response and increase response rates. The MBT-2 model appears relevant to investigate immunological BCa sex differences.

**Patient summary:**

Our studies suggest that combining antiandrogen treatments with BCa immunotherapy may improve response rates in men. We also demonstrate the utility of the MBT-2 mouse model to study sex differences in BCa.

## Take Home Message

These preclinical studies in mice using the MBT-2 model of bladder cancer suggests that the combination of antiandrogens and immunotherapy for non–muscle-invasive bladder cancer may improve response rates.

## 1 Introduction

Bladder cancer (BCa) is the seventh most common cancer worldwide [1]. Men are three to four times more likely to be diagnosed than women [2], who often have more aggressive tumors at diagnosis [3]. At diagnosis, over 70% of tumors are classified as non–muscle-invasive bladder cancer (NMIBC). Intravesical bacillus Calmette-Guerin (BCG) is commonly used to decrease the risk of recurrence of high-grade NMIBC [4]. Nonetheless, approximately 35% of patients who receive BCG still have recurrences [5]. More recently, inhibition of programmed cell death protein-1 (PD-1) or programmed cell death-ligand 1 (PD-L1) has emerged as effective BCa immunotherapeutic treatment [6], [7]. However, only 20–30% of patients have a significant response to inhibition of these immune checkpoints [8]. These incomplete response rates to both BCG and checkpoint inhibitors highlight the need for novel approaches to improve response rates.

The importance of sex and hormonal differences on BCa treatment response remains relatively unexplored. Large contemporary series do not suggest sex differences in response to BCG treatment reported in prior series [9], [10]. Sociological, health system, and presentation differences nonetheless confound the assessment of biological treatment differences [11]. Existing studies suggest that hormonal differences may impact outcomes, with estrogens being potentially protective against BCa development but possibly supportive of BCa progression [12]. Androgens and the androgen receptor (AR) are more robustly implicated in bladder carcinogenesis and BCa progression [13]. Additionally, androgens are recognized to have immunosuppressive properties [14], which likely contribute to sex differences in various pathologies [15]. Recent studies have found AR-suppressive therapy associated with improved BCa outcomes, highlighting the potential for clinical impact [16].

In this study, we assessed the use of AR antagonism as an approach to improve the antitumor response induced by immunotherapy. We first demonstrate how the MBT-2 BCa murine model recapitulates human biology of NMIBC, including sex differences. We use the MBT-2 model to evaluate the AR antagonist enzalutamide in combination with BCG or anti–PD-1 immunotherapy.

## 2 Patients and methods

### 2.1 Cell culture

MBT-2 cells were kindly provided by Dr. Michael O’Donnell (University of Iowa, IA, USA) and were cultured at 37 °C with 5% CO_2_ in RPMI-1640 medium (Wisent, St-Bruno, Quebec, QC, Canada) supplemented with 10% inactivated fetal bovine serum (FBS; Wisent). Mycoplasma testing was performed periodically and before injection into mice.

### 2.2 Animal experiments

Adult C3H/He mice (Charles River, St-Constant, QC, Canada) aged 6–8 week were housed with water and food *ad libitum*. To produce tumors, 2 × 10^5^ MBT-2 cells in 100 μl of Hank’s balanced salt solution (HBSS, Wisent) were injected subcutaneously on each flank. Tumor size was monitored biweekly using calipers, with tumor volume calculated by the following formula: *V* = $\frac{{\mathrm{width}}^{2}\times length}{2}$. Mice were sacrificed when the tumor volume reached 2 cm^3^, with tumors freshly dissociated or snap frozen for future experiments.

#### 2.2.1 Experimental design

In a first experiment to compare NMIBC with MBT-2 tumors, mice received no treatment. In a second experiment, mice received intraperitoneal injections of 200 μg of rat anti–PD-1 monoclonal antibody (mAb, clone RPM1-14) or isotype control (clone 2A3, both from BioXcell Inc, West Lebanon, NH, USA) on days 3, 5, 7, and 9 after tumor cell injection and/or 10 mg/kg of oral gavage with enzalutamide (100 μl; MedChemExpress, Monmouth Junction, NJ, USA) or vehicle (5% methylcellulose, 1% tween80) three times a week. In a third experiment, mice received weekly intratumoral injections of BCG, 1 × 10^6^ colony-forming unit/25 μl/tumor site and poly(I:C) (2.5 μg/25 μl/tumor site) and/or 10 mg/kg of enzalutamide (MedChemExpress) and/or vehicle gavage until complete tumor regression or sacrifice. We previously reported that the addition of poly(I:C) to BCG treatment in this model recapitulates clinical response rates [17]. For long-term immune memory response evaluation, mice with complete responses and naïve control mice were challenged by subcutaneous injection of 2 × 10^5^ MBT-2 cells 80 day after the primary tumor cell implantation.

### 2.3 Tumor dissociation

After excision, tumors were minced into 2–4 mm^2^ fragments and mechanically disrupted using a GentleMACs dissociator (Miltenyi Biotec, San Diego, CA, USA). Dissociated tumors were incubated with 1500 collagenase digestive units of collagenase I (Sigma-Aldrich, Oakville, ON, Canada) and 5 mU/ml of dispase II (Roche Diagnostics, Meylan, France) during 20 min of agitation at 37 °C. A 70 μm cell strainer was used to remove clumps, and red blood cells were lysed with ammonium chloride potassium (1.5 M NH_4_Cl, 100 mM KHCO_3_, and 100 mM ethylenediaminetetra-acetic acid [EDTA]) with gentle shaking at 4 °C for 5 min prior to a final wash with HBSS. Human tumors were processed using EMEM media (Wisent), while murine tumors were processed in RPMI media (Wisent).

### 2.4 Cell staining and flow cytometry analyses

#### 2.4.1 Murine cells

Cells were incubated with purified rat anti-mouse CD16/CD32 Fc block (BD Biosciences, Mississauga, Ontario, Canada) for 15 min at 4 °C to reduce nonspecific staining. After washing with buffer containing 3% FBS, 1 mM EDTA, and 0.09% NaNO3, cellular subpopulations were identified with fluorescent dye-conjugated mAb. For murine cells, three antibody panels were incubated for 30 min at 4 °C in the dark. Antibodies used are detailed in Supplementary Table 1.

#### 2.4.2 Human cells

Frozen dissociated NMIBC samples obtained from 15 patients (CHU de Québec-Université Laval ethics approval #2012-253 and 2017-2749) were analyzed by flow cytometry. Cells were first incubated with human Fc block (#564219; BD Biosciences) for 15 min at 4 °C and then stained for 30 min at 4 °C in the dark with an antibody panel. Antibodies used are detailed in Supplementary Table 2.

For both human and murine cells, stained cells were analyzed using the BD LSRFortessa cytometer (BD Biosciences, San Jose, CA, USA). Data were processed using BD FACS Diva software (BD Biosciences), with analysis using FlowJo software (version 10.7.1; Flowjo, LLC, Ashland, OR, USA). Cell viability was assessed using a viability stain (FVS-780; BD Biosciences, or FVS-450; BD Biosciences), and doublets were excluded based on forward scatter-H against forward scatter-W. Compensation controls were done using compensation beads (BD CompBeads; BD Biosciences) and fluorescence minus one controls on fresh samples.

### 2.5 RNA sequencing

RNA was isolated from murine tumor tissues using the Invitrogen mirVana miRNA isolation kit (Fisher Scientific, Hampton, NH, USA) according to the manufacturer’s instructions.

DNase I (RNeasy MinElute Cleanup kit; QIAGEN, Montréal, QC, Canada) was used to remove genomic DNA contamination, while RNA integrity was assessed with the RNA 6000 pico assay (Agilent Technologies, Santa Clara, CA, USA). The minimum RNA integrity number across all samples was 8.7. RNA sequencing was performed by the CHU de Québec-Université Laval Research Center genomics platform. Briefly, samples were processed using NEBNext Ultra II directional RNA library prep kit (New England Biolabs, Whitby, ON, Canada), with library preparations sequenced on the Novaseq 6000 Sequencing System (Illumina, San Diego, CA, USA) with 100 base-pair single end reads, resulting in at least 25 million single end reads per sample. Following a previously described pipeline [18], differential gene expression and CIBERSORTx analysis using a mouse-specific signature to calculated relative immune cell fractions were based on transcripts per million counts [19], [20].

### 2.6 Statistical analyses

Data are presented as means ± standard error of mean. Student *t* test and one-way analysis of variance compared means between groups. Normality for patient biopsies was evaluated by both D’Agostino-Pearson and Shapiro-Wilk normality tests. GraphPad Prism 8.0 (Graphpad Software, San Diego, CA, USA) was used for all statistical tests.

## 3 Results

### 3.1 Immune composition of the MBT-2 model recapitulates human NMIBC, including sex differences

**Fig. 1:**
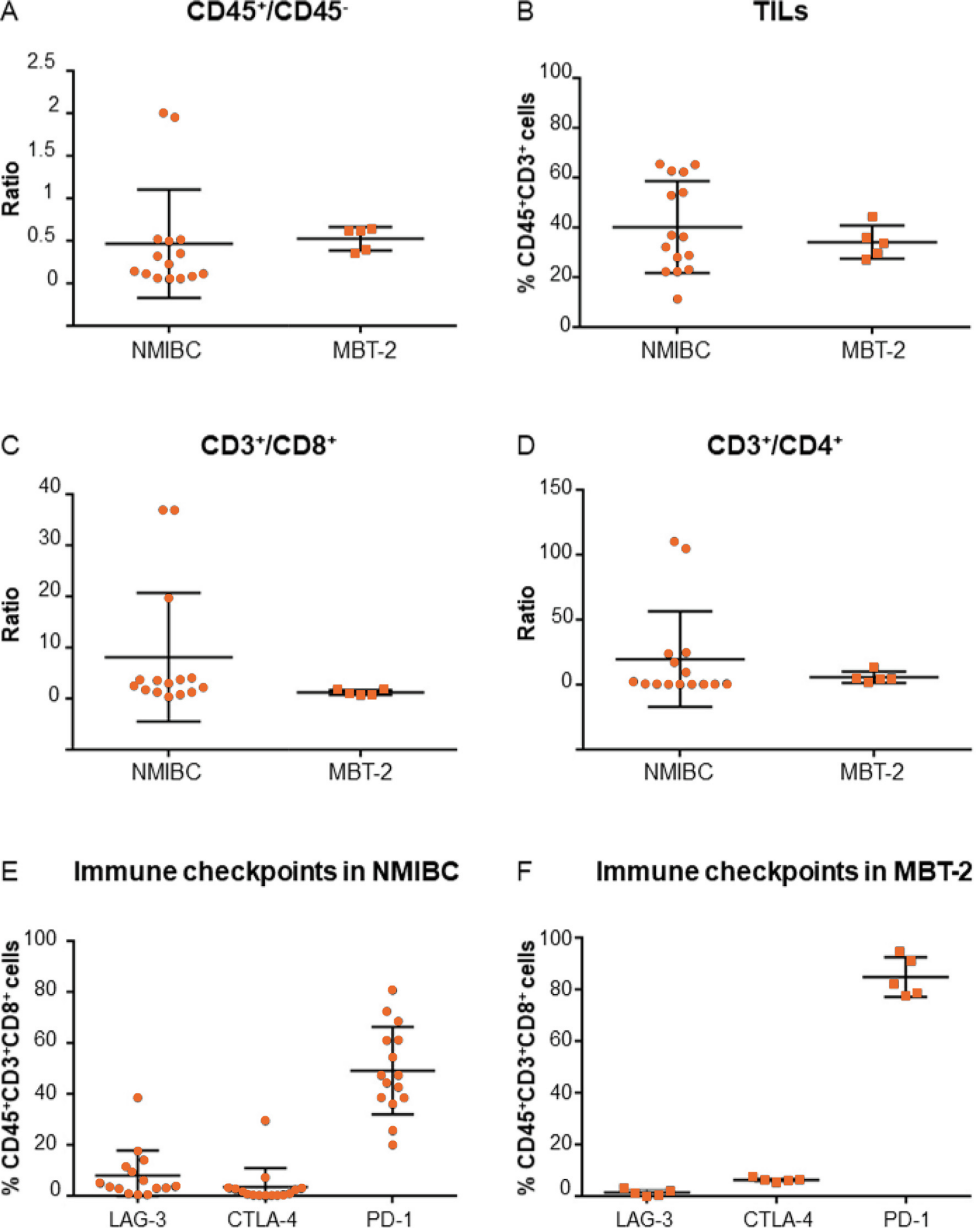
MBT-2 tumors have similar immune composition to that of NMIBC specimens. Immune compositions of MBT-2 tumors and human NMIBC specimens have been compared by flow cytometry analyses. (A) MBT-2 tumors have similar CD45^+^/CD45^–^ ratio to patient’s tumors. (B) The proportion of CD45^+^CD3^+^ cells (TILs) is similar between patient’s tumors and MBT-2 tumors. (C) The CD3^+^/CD8^+^ ratio is similar between patient’s tumors and MBT-2 tumors. (D) The CD3^+^/CD4^+^ ratio is similar between patient’s tumors and MBT-2 tumors. (E) Expression of immune checkpoint markers on CD45^+^CD3^+^CD8^+^ cells in NMIBC specimens. (F) Expression of immune checkpoint markers on CD45^+^CD3^+^CD8^+^ cells in MBT-2 tumors. NMIBC = non–muscle-invasive bladder cancer; PD-1 = programmed cell death protein-1; TIL = tumor-infiltrating lymphocyte.

We compared the immune composition of murine MBT-2 tumors and patient NMIBC using flow cytometry. First, we observed that the proportion of immune cells (using the CD45^+^/CD45^–^ ratio) is similar between human NMIBC and MBT-2 murine tumors (Fig. 1A). The proportion of tumor-infiltrating lymphocytes (TILs; CD45^+^CD3^+^ cells; Fig. 1B), CD3^+^/CD8^+^ ratio (Fig. 1C), and CD3^+^/CD4^+^ ratio (Fig. 1D) were also similar. Finally, the expression of the immune checkpoint markers LAG-3, CTLA-4, and PD-1 on CD8^+^ T cells (CD45^+^CD3^+^CD8^+^ cells) was comparable between NMIBC and MBT-2 tumors (Fig. 1E and 1F).

**Fig. 2:**
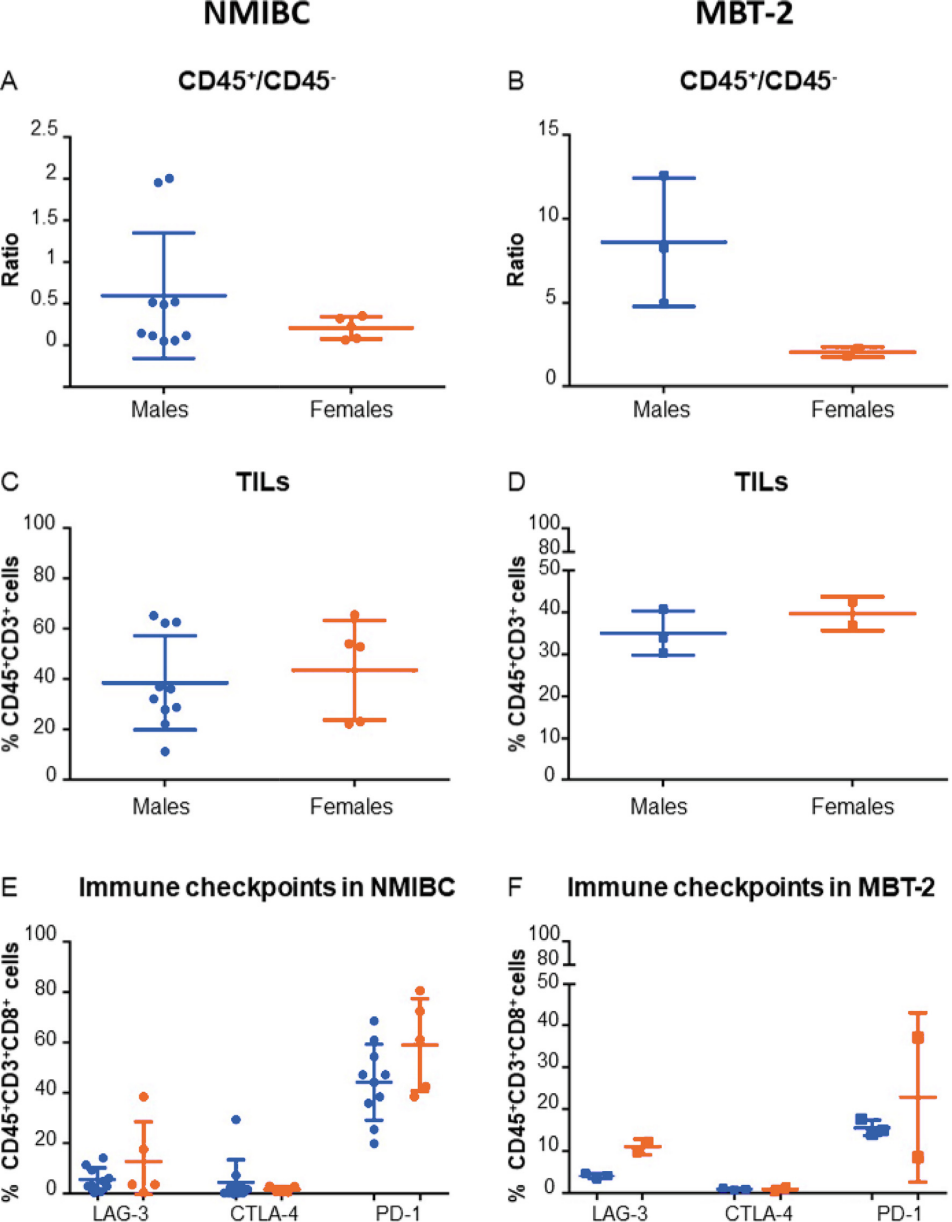
Our syngeneic MBT-2 murine model reproduces BCa sex differences observed in NMIBC patients. Immune compositions of MBT-2 tumors and human NMIBC specimens have been compared by flow cytometry analyses. (A) Comparison of the CD45^+^/CD45^–^ ratio in NMIBC specimens from female and male patients. (B) Comparison of the CD45^+^/CD45^–^ ratio in MBT-2 tumors from female and male mice. (C) Comparison of the proportion of CD45^+^CD3^+^ cells (TILs) in NMIBC specimens from female and male patients. (D) Comparison of the proportion of CD45^+^CD3^+^ cells (TILs) in MBT-2 tumors from female and male mice. (E) Comparison of the expression of immune checkpoints on CD45^+^CD3^+^CD8^+^ cells in NMIBC specimens from female and male patients. (F) Comparison of the expression of immune checkpoints on CD45^+^CD3^+^CD8^+^ cells in MBT-2 tumors from female and male mice. BCa = bladder cancer; NMIBC = non–muscle-invasive bladder cancer; PD-1 = programmed cell death protein-1; TIL = tumor-infiltrating lymphocyte.

Next, we compared differences in the immune composition tumors between sexes in NMIBC and MBT-2 tumors (Fig. 2). We observed sex differences in the ratio of CD45^+^/CD45^–^ cells ratio (Fig. 2A and 2B). This was also observed for TILs (Fig. 2C and 2D) and the expression of the immune checkpoint markers LAG-3, CTLA-4, and PD-1 (Fig. 2E and 2F). These observed differences between tumors from male versus female mice together recapitulate those observed in NMIBC patients and reported differences in human tumors in the literature [21]. We did not find these differences in the commonly used MB49 murine model of BCa (data not shown). Together, these data suggest that the MBT-2 murine model is relevant for the study of sex differences in the immune tumor microenvironment of BCa.

### 3.2 Combination of enzalutamide and BCa immunotherapy synergizes to improve the therapeutic response in male MBT-2 mice

Using different BCa cell lines, we next assessed whether AR antagonists inhibit proliferation *in vitro* and observed that AR antagonists decrease the proliferation of human and murine BCa cells, principally in lower-grade BCa cells (Supplementary Fig. 1). These results also confirmed the choice of enzalutamide for use in *in vivo* studies.

**Fig. 3:**
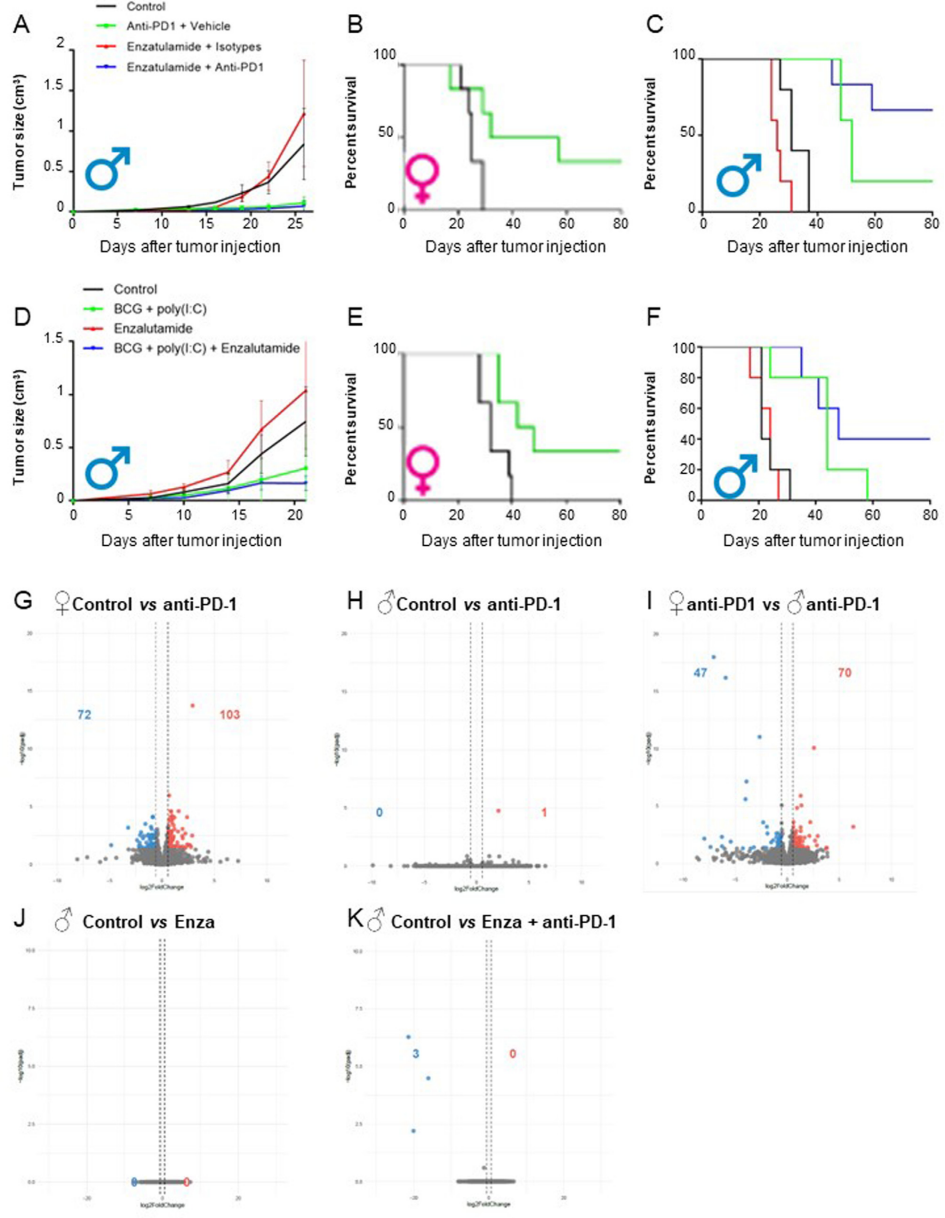
Combination of enzalutamide and immunotherapies (anti–PD-1 or BCG + poly[I:C]) improves survival in male mice. All treatment groups were composed of six mice. (A) Anti–PD-1 alone and in combination with enzalutamide decrease tumor growth over time in male mice. (B) Anti–PD-1 therapy increases the survival of female mice. (C) Anti–PD-1 alone increases the survival of male mice, but combination with enzalutamide increases the survival of mice even more. (D) BCG + poly(I:C) treatment alone and in combination with enzalutamide decrease tumor growth over time in male mice. (E) BCG + poly(I:C) treatment increases female mice survival. (F) BCG + poly(I:C) treatment increases the survival of male mice, but combination with enzalutamide increases the survival of mice even more. Volcano plots show the results of tumor RNA sequencing and differential gene expression analyses comparing tumors from the (G) control group (*n* = 3) versus anti–PD-1 group (*n* = 3) in female mice, (H) control group (*n* = 3) versus anti–PD-1 group (*n* = 3) in male mice, (I) anti–PD-1 group (*n* = 3) in female mice versus anti–PD-1 group (*n* = 3) in male mice, (J) control group (*n* = 3) versus enzalutamide group in male mice (*n* = 3), and (K) control group (*n* = 3) versus enzalutamide + anti–PD-1 group (*n* = 2) in male mice. BCG = bacillus Calmette-Guerin; ENZA = enzalutamide; PD-1 = programmed cell death protein-1.

Using the immunocompetent and syngeneic MBT-2 murine model, we first observed that treatment with anti–PD-1 mAb decreased tumor growth and resulted in a complete response in typically one out of six male mice, about half the number observed in female mice (Fig. 3A–C). We also showed that the survival in response to PD-1 inhibition in female mice was superior to that observed in male mice (Fig. 3B and C). To assess the effect of AR antagonism on survival, we treated male mice with enzalutamide. Treatment with enzalutamide alone resulted in tumor growth and survival similar to control (Fig. 3C). Subsequently, we assessed the combination of enzalutamide and anti–PD-1 mAb. We observed that the combination resulted in a significant increase in the survival of male mice as well as greater tumor growth inhibition compared with either monotherapy (Fig. 3A–C). Notably, the combination of enzalutamide and anti–PD-1 mAb increased the proportion of complete responses. A rechallenge with reinoculation of MBT-2 cells was performed in the two available surviving mice, with no tumor growth observed indicating immune memory.

To confirm the therapeutic potential of enzalutamide to improve BCa immunotherapy, we further assessed the treatment with the combination of enzalutamide and BCG + poly(I:C) in the MBT-2 murine model. As expected, BCG + poly(I:C) treatment in male mice improved survival and decreased tumor growth compared with control, while no benefit was seen with enzalutamide alone (Fig. 3D–F). Similar to responses with anti–PD-1 mAb, BCG + poly(I:C) treatment was superior in female mice to that in male mice (Fig. 3E and F). However, the combination of BCG + poly(I:C) and enzalutamide showed the highest tumor growth inhibition (Fig. 3D) and survival (Fig. 3F). Immune memory was also demonstrated, with no tumors observed growing in both mice treated with the combination of BCG + poly(I:C) and enzalutamide that were rechallenged. Importantly, a comparison between male and female mice highlights that the addition of enzalutamide improves the response rate for both BCG + poly(I:C) and PD-1 inhibition in male mice to resemble the superior results obtained in females (Fig. 3A–F).

**Fig. 4:**
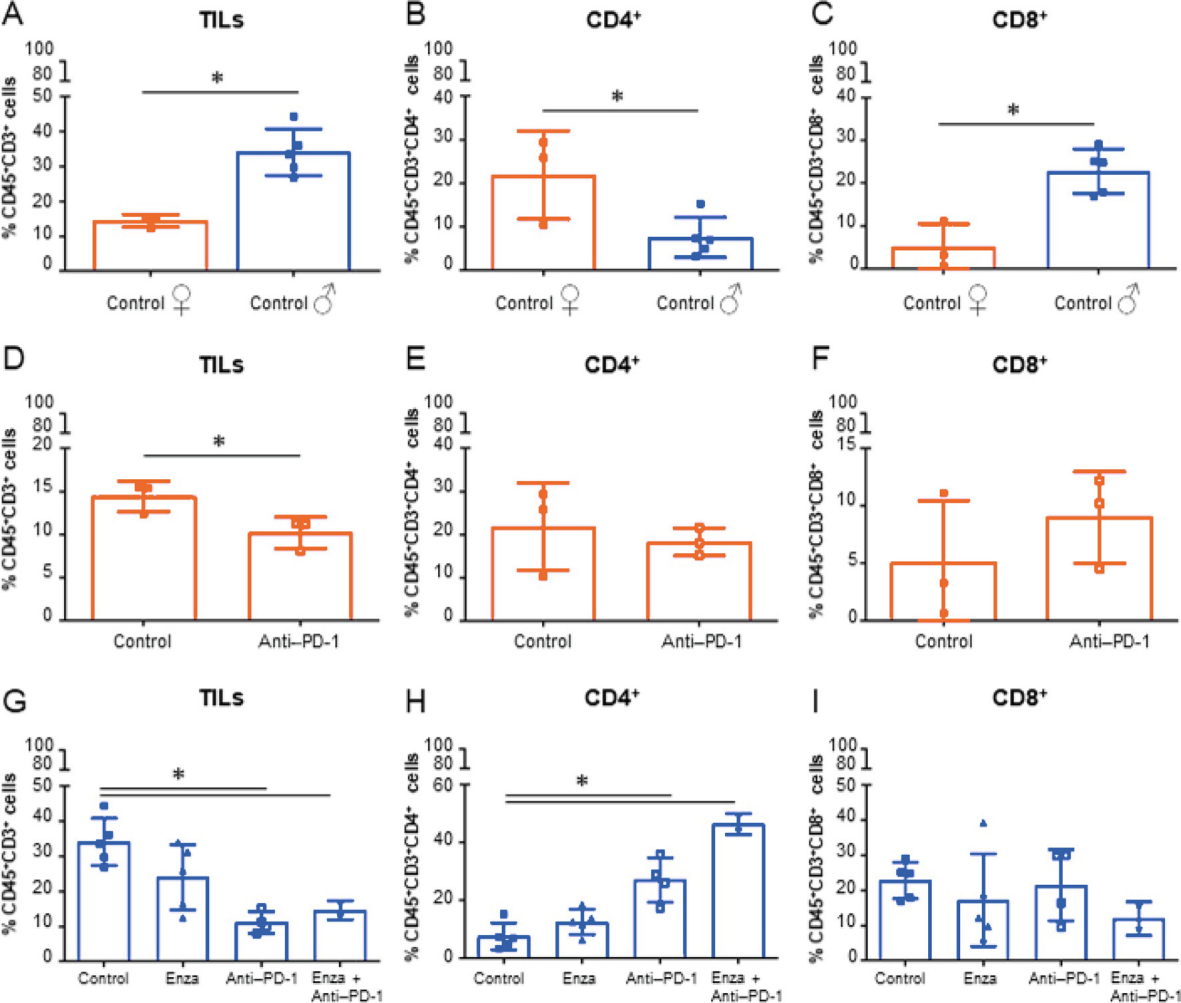
Anti–PD-1 treatment and combination of anti–PD-1 treatment + enzalutamide decrease TILs in tumors mice. The MBT-2 model has been used in female and male mice to study immune composition of tumors by multicolor flow cytometry analyses. (A) Tumors in male mice have significantly more TILs (CD45^+^CD3^+^ cells) than those in female mice (*p = 0.003). (B) Tumors in male mice have significantly fewer CD4^+^ T cells (CD45^+^CD3^+^CD4^+^ cells) than those in female mice (*p = 0.0303). (C) Tumors in male mice have significantly more CD8^+^ T cells (CD45^+^CD3^+^CD8^+^ cells) than those in female mice (*p = 0.0037). (D) Anti–PD-1 alone significantly decreases TILs in female mice (*p = 0.0443). (E) Anti–PD-1 alone does not seem to affect CD4^+^ T cells in female mice. (F) Anti–PD-1 treatment does not seem to affect CD8^+^ T cells in female mice. (G) Anti–PD-1 alone (*p = 0.0127) and in combination with enzalutamide (*p = 0.0004) significantly decrease TILs in male mouse tumors. (H) Anti–PD-1 alone (*p = 0.0001) and in combination with enzalutamide (*p = 0.0021) significantly increase CD4^+^ T cell infiltration in male mouse tumors. (I) Anti–PD-1 alone and in combination with enzalutamide does not seem to impact CD8^+^ T cell infiltration in male mouse tumors. ENZA = enzalutamide; PD-1 = programmed cell death protein-1; TIL = tumor-infiltrating lymphocyte.

Differential gene expression on collected tumors shows an important difference between control and anti–PD-1 treatment among female mice but not among male mice (Fig. 3G and H). Interestingly, female mice receiving anti–PD-1 showed greater differential gene expression changes than the male anti–PD-1 group (Fig. 3I). However, minimal changes were observed with enzalutamide or the combination of enzalutamide and anti–PD-1, suggesting that the observed synergy was not mechanistically related to gene expression changes. We therefore further investigated the immune composition of the same tumors using flow cytometry. We first observed that tumors from the male control group have significantly more TILs and CD8^+^ T cells, and significantly fewer CD4^+^ T cells (CD45^+^CD3^+^CD4^+^ cells) than female controls (Fig. 4A–C). Anti–PD-1 treatment significantly decreased TIL infiltration in tumors of female and male mice, but did not impact the proportion of CD4^+^ and CD8^+^ T cells in females, whereas in males, a significant increase of CD4^+^ T cells is observed (Fig. 4D–I). Notably, the therapeutic combination of enzalutamide and anti–PD-1 induces a decrease of TILs but an increase of the proportion of CD4^+^ T cells in tumors (Fig. 4G and H).

**Fig. 5:**
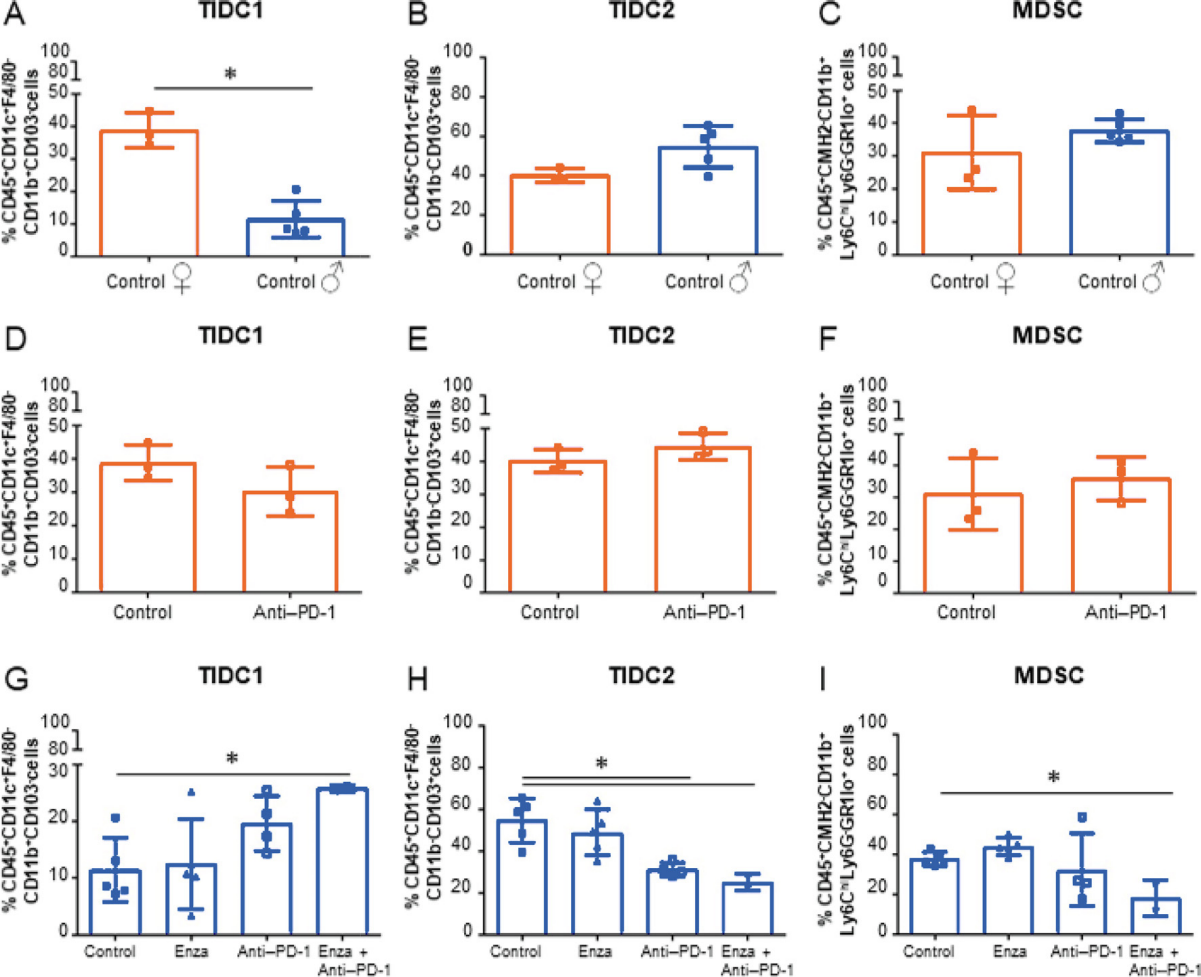
The combination of enzalutamide and anti–PD-1 treatment promote proinflammatory profile in tumors in mice. The MBT-2 model has been used in female and male mice to study immune composition of tumors by multicolor flow cytometry analyses. (A) Tumors in male mice have significantly fewer type 1 tumor-infiltrating dendritic cells (TIDC1; CD45^+^CD11c^+^F4/80^–^CD11b^+^CD103^–^ cells) than those in female mice (*p = 0.0037). (B) Tumors in male mice seem to have more of type 2 tumor-infiltrating dendritic cells (TIDC2; CD45^+^CD11c^+^F4/80^-^CD11b^–^CD103^+^ cells) than those in female mice. (C) Tumors in male and female mice have similar proportions of myeloid-derived suppressor cells (MDSC; CD45^+^CMH2^+^CD11b^+^Ly6C^High^Ly6G^–^GR1^Low^ cells). Anti–PD-1 treatment alone does not seem to affect the proportions of (D) TIDC1, (E) TIDC2, or (F) MDSC in female mouse tumors. (G) Combination of anti–PD-1 and enzalutamide treatments significantly increase the proportion of TIDC1 (*p = 0.019). (H) Anti–PD-1 alone (*p = 0.004) and in combination with enzalutamide (*p = 0.014) significantly decrease the proportion of TIDC2. (I) Combination of anti–PD-1 and enzalutamide treatments significantly decreases the population of MDSC (*p = 0.006). ENZA = enzalutamide; PD-1 = programmed cell death protein-1; TIL = tumor-infiltrating lymphocyte.

For innate immune cells, we observed a significant decrease of tumor-infiltrating dendritic cells type 1 (TIDC1; CD45^+^CD11c^+^F4/80^–^CD11b^+^CD103^–^ cells; Fig. 5A) and type 1 tumor-infiltrating macrophages (M1, CD45^+^CMH2^+^F4/80^+^CD11c^–^CD11b^+^ cells; Supplementary Fig. 2), and a significant increase of M2 (CD45^+^CMH2^+^F4/80^+^CD11c^+^CD11b^+^ cells; Supplementary Fig. 2) in male control mice compared with female control mice. No significant differences were observed in female mice treated with anti–PD-1 compared with female control mice. The combination of enzalutamide and anti–PD-1 treatment significantly increased TIDC1 cells and significantly decreased both tumor-infiltrating dendritic cells type 2 (TIDC2; CD45^+^CD11c^+^F4/80^–^CD11b^–^CD103^+^) cells and myeloid-derived suppressor cells (MDSCs; CD45^+^CMH2^+^CD11b^+^Ly6C^High^Ly6G^–^GR1^Low^) cells compared with either monotherapy or controls (Fig. 4G–I).

## 4 Discussion

While differences between sexes in BCa are well known, application of these differences to treatment strategies has yet to be made. Our results suggest the potential to use AR antagonists in combination with immunotherapies in males to improve treatment response rates. We also demonstrate key aspects of the MBT-2 mouse model, highlighting how it is comparable with the immune composition of NMIBC tumors from patients and sex differences reported in the literature.

Our results provide particular support to combine antiandrogens with BCG treatment for NMIBC. We demonstrate notable similarities from the MBT-2 model to NMIBC in patients, which importantly include sex differences in the immune infiltrate. Clinically, there is limited evidence for AR antagonists for BCa, although accumulating data available support efficacy for 5-alpha reductase inhibitors [22]. Nonetheless, given the dose-response rates observed in vitro, it remains plausible that the more potent AR antagonism may induce greater synergy, as seen in our murine experiments.

Our *in vivo* results demonstrating synergy with AR antagonism and BCG or anti–PD-1 mAb suggest a broader mechanism not specific to either immunotherapy. Initial clinical results combining pembrolizumab and enzalutamide in prostate cancer suggest similar synergy [23], with a recent study suggesting that androgens play a role in suppressing T-cell function [24] important for response to immune checkpoint inhibitors. As the composition of the immune cell infiltrate in the BCa microenvironment is critical for the response to both immune checkpoint inhibitors and BCG, differences at these levels may explain the synergy, consistent with our flow cytometry and RNA-sequencing studies. Indeed, the accumulation of immunosuppressive innate immune cells portends a worse prognosis and is also associated with a poorer response to BCG [25], [26], [27]. Consistent with this literature, we found that the combination of enzalutamide and anti–PD-1 treatment increased proinflammatory TIDC1 and decreased anti-inflammatory TIDC2 and MDSCs in male MBT-2 tumors. The low AR expression in MBT-2 cells (data not shown) also supports an indirect mechanism acting on the immune microenvironment and helps explain why enzalutamide alone had no benefit. Nonetheless, our tumor analyses were performed on tumors remaining after treatment; it is possible that the differences observed between control and treated female mice reflect an increased immunoregulatory response due to treatment selection. For the male mice, the less effective immune response to tumors and the fewer differentially expressed genes may represent tumors that resemble more MBT-2 tumors, which have less selection pressure from the immune system. In this case, the changes we observed with the combination of enzalutamide and anti–PD-1 immunotherapy may reflect greater selection pressure on tumors. In either case, changes to the immune composition are much more pronounced than gene expression changes, reflecting the importance of understanding changes in the immune tumor microenvironment to improve immunotherapy response rates.

Our study has several limitations. Owing to sample availability, RNA sequencing included only two tumors in the male combination group. The collection of tumors at maximal tumor size may also obscure changes induced at the transcriptional level. Another limitation is the low number of mice per group for each animal study. Finally, additional mechanistic possibilities including epigenetic or unmeasured immune cell populations not evaluated may provide further insights.

## 5 Conclusions

In summary, our preclinical studies suggest that AR antagonism may synergize to improve BCa immunotherapy response rates in men through modulation of the immune cell composition of tumors. Translation of our results to patients is facilitated by the large urological experience with AR antagonists, with a phase II trial of bicalutamide in men receiving BCG underway (NCT05327647).

  ***Author contributions*:** Paul Toren had full access to all the data in the study and takes responsibility for the integrity of the data and the accuracy of the data analysis.

*Study concept and design*: Bergeron, Fradet, Toren.

*Acquisition of data*: Gris, Besançon, Joncas, Picard.

*Analysis and interpretation of data*: Gris, Besançon, Joncas, Picard.

*Drafting of the manuscript*: Gris, Besançon.

*Critical revision of the manuscript for important intellectual content*: Bergeron, Fradet, Toren.

*Statistical analysis*: Gris, Besançon, Toren.

*Obtaining funding*: Bergeron, Fradet, Toren.

*Administrative, technical, or material support*: Gris, Joncas, Picard.

*Supervision*: Bergeron, Fradet, Toren.

*Other*: None.

  ***Financial disclosures:*** Paul Toren certifies that all conflicts of interest, including specific financial interests and relationships and affiliations relevant to the subject matter or materials discussed in the manuscript (eg, employment/affiliation, grants or funding, consultancies, honoraria, stock ownership or options, expert testimony, royalties, or patents filed, received, or pending), are the following: Alain Bergeron reports research funding from Astellas, IMV Inc, and GSK Biologicals, as well as personal fees as a consultant from Merck. Yves Fradet reports research funding from Tersera, Astellas, and IMV Inc, as well as personal fees as a consultant from Merck, Sanofi, Ferring, Amgen, Janssen, and Astellas. Paul Toren reports research funding from Bristol-Myers-Squibb, Sanofi, and Janssen, as well as personal fees as a consultant from Bayer, Ferring, TerSera, Janssen, Sanofi, and Abbvie. The other authors have no conflicts of interest to declare.

  ***Funding/Support and role of the sponsor*:** This work was supported by the Canadian Cancer Society Research Institute (Grant #701601; Yves Fradet) and by the Cancer Research Society (Upcycle Program; Alain Bergeron, Yves Fradet, and Paul Toren). Paul Toren is supported by a clinician-scientist award from Fonds de Recherche du Québec – Santé (#32774).

  ***Acknowledgments*:** We thank the CHU de Québec-Université Laval Research Center genomics and pathology platforms for their help with RNA sequencing and human tumor processing. We also acknowledge Julien Prunier from the laboratory of Dr. Arnaud Droit for his help with bioinformatics analysis.

## Figures and Tables

**Supplementary figure 1:**