# Supplementary material for: Combining Antiandrogens with Immunotherapy for Bladder Cancer Treatment
Source: Eur Urol Open Sci. 2022 Jul 26;43:35–44. doi: 10.1016/j.euros.2022.06.007 (PMC9557088; doi:10.1016/j.euros.2022.06.007)
Supplement: Supplementary data 1 [file mmc1.docx]

**Supplementary material**

**Supplementary Figure 1: Anti-androgen therapies suppress BCa cells proliferation.**

Murine (a-b) and human (c-f) BCa cell lines were seeded at 5000 cells per well and treated with various concentrations (0 to 40 μM) of enzalutamide, VT-464 or R1181 (0.1 nM, negative control) for 96 h at 37°C. a) MBT-2 cells proliferation decrease seems to be proportional to the doses of anti-androgens therapies (enzalutamide and VT-464) and is significant at 20 μM (p*˂0.001), 40 μM (p*˂0.001) of enzalutamide and 40 μM (p*=0.0013) of VT-464. b) MB49 cells proliferation decrease seems to be proportional to the doses of anti-androgens therapies and is significant for at 40 μM of enzalutamide (p*=0.007). c) RT4 cells proliferation decrease seems to be proportional to the doses of anti-androgens therapies and is significant at 40 μM of VT-464 (p*=0.003). d) VM-CUB-1 cells proliferation decrease seems to be proportional to the doses of anti-androgens therapies and is significant at 40 μM of VT-464. (p*˂0.001). e) T24 cells proliferation decrease seems to be proportional to the doses of anti-androgens therapies. f) 639V cells proliferation decrease seems to be proportional to the doses of anti-androgens therapies.

**Supplementary Figure 2: Anti-androgen therapies suppress BCa cells proliferation.**

MBT-2 model has been used in female and male mice to study immune composition of tumors by multicolor flow cytometry analyses. Male mice tumors have significantly more of type 1 tumor infiltrating macrophages (TAM M1, CD45^+^CMH2^+^F4/80^+^CD11c^-^CD11b^+^ cells, p*=0.0021) and significantly more of TAM M2 (CD45^+^CMH2^+^F4/80^+^ CD11c^+^CD11b^+^ cells, p*=0.0127) than female.

**Supplementary Table 1:** Mouse antibodies for flow cytometry analyses.

| Antibody | Origin | Reactivity | Fluorochrome | Provider |
| --- | --- | --- | --- | --- |
| Ly6-C | Rat | Mouse | Pe-Cy7 | BD Biosciences, #560593 |
| CMH2 | Rat | Mouse | BV605 | Biolegend, #107621 |
| CD45 | Rat | Mouse | V500 | BD Biosciences, #561487 |
| CD11b | Rat | Mouse | PerCPCy5.5 | BD Biosciences, #561114 |
| GR1 | Rat | Mouse | AF700 | BD Biosciences, #557979 |
| Ly6G | Rat | Mouse | APC-Cy7 | Biolegend, #127623 |
| F4/80 | Rat | Mouse | FITC | Biolegend, #123107 |
| CD103 | Hamster | Mouse | PeCy7 | Biolegend, #121425 |
| CD11c | Hamster | Mouse | APC-Cy7 | BD Biosciences, #561241 |
| CD3e | Hamster | Mouse | APC-Cy7 | BD Biosciences, #557596 |
| CD4 | Rat | Mouse | FITC | BD Biosciences, #557307 |
| CD8a | Rat | Mouse | BV605 | BD Biosciences, #563152 |
| TIM3 | Rat | Mouse | AF647 | Biolegend, #134005 |
| PD-1 | Hamster | Mouse | PeCF594 | BD Biosciences, #562523 |
| CTLA-4 | Hamster | Mouse | PE | BD Biosciences, #561718 |

**Supplementary Table 2:** Human antibodies for flow cytometry analyses.

| Antibody | Origin | Reactivity | Fluorochrome | Provider |
| --- | --- | --- | --- | --- |
| CD45 | Mouse | Human | V500 | BD Biosciences, #560367 |
| CD3 | Mouse | Human | APC-Cy7 | BD Biosciences, #557832 |
| CD4 | Mouse | Human | FITC | BD Biosciences, #561005 |
| CD8 | Mouse | Human | BV786 | BD biosciences, #563824 |
| CTLA-4 | Mouse | Human | PE | BD Biosciences, #560939 |
| LAG-3 | Mouse | Human | PerCP Cy5.5 | eBioscience,  #46-2239-42 |
| PD-1 | Mouse | Human | PeCF594 | BD Biosciences, #565024 |
